# Partial Autocorrelation Diagnostics for Count Time Series

**DOI:** 10.3390/e25010105

**Published:** 2023-01-04

**Authors:** Christian H. Weiß, Boris Aleksandrov, Maxime Faymonville, Carsten Jentsch

**Affiliations:** 1 Department of Mathematics and Statistics, Helmut Schmidt University, 22043 Hamburg, Germany; 2 Department of Statistics, TU Dortmund University, 44221 Dortmund, Germany

**Keywords:** autoregressive model, count time series, INAR bootstrap, partial autocorrelation function, Yule–Walker equations

## Abstract

In a time series context, the study of the partial autocorrelation function (PACF) is helpful for model identification. Especially in the case of autoregressive (AR) models, it is widely used for order selection. During the last decades, the use of AR-type count processes, i.e., which also fulfil the Yule–Walker equations and thus provide the same PACF characterization as AR models, increased a lot. This motivates the use of the PACF test also for such count processes. By computing the sample PACF based on the raw data or the Pearson residuals, respectively, findings are usually evaluated based on well-known asymptotic results. However, the conditions for these asymptotics are generally not fulfilled for AR-type count processes, which deteriorates the performance of the PACF test in such cases. Thus, we present different implementations of the PACF test for AR-type count processes, which rely on several bootstrap schemes for count times series. We compare them in simulations with the asymptotic results, and we illustrate them with an application to a real-world data example.

## 1. Introduction

*Autoregressive (AR) models* for time series date back to Walker [1], Yule [2], and they assume the current observation of the considered process to be generated from its own past by a linear scheme. The ordinary pth-order AR-model for a real-valued process ${\left(Z_{t}\right)}_{t\in Z=\{\ldots ,-1,0,1,\ldots \}}$, abbreviated as AR(p) model, is defined by the recursive scheme

(1) $$Z_{t}\;=\;\alpha_{1}\cdot Z_{t-1}+\ldots +\alpha_{p}\cdot Z_{t-p}+\varepsilon_{t}\;\left(\alpha_{p}\neq 0\right),$$

where the innovations ${\left(\varepsilon_{t}\right)}_{Z}$ are independent and identically distributed (i. i. d.) real-valued random variables (rv), which are also assumed to be square-integrable (“white noise”). To ensure a (weakly) stationary and causal solution for the AR(p) recursion (1), the AR-parameters $\alpha_{1},\ldots ,\alpha_{p}\in R$ have to be chosen such that the roots of the characteristic polynomial $\alpha \left(z\right)=1-\alpha_{1}\;z-\ldots -\alpha_{p}\;z^{p}$ are outside the unit circle. Then, if the innovations ${\left(\varepsilon_{t}\right)}_{Z}$ follow a normal distribution, also the observations ${\left(Z_{t}\right)}_{Z}$ are normal, leading to the Gaussian AR(p) process.

A characteristic property of the AR(p) process is given by the fact that its autocorrelation function (ACF), $\rho \left(h\right)=Corr\left[Z_{t},Z_{t-h}\right]$ with h∈N={1,2,…} and ρ(0)=1, satisfies the following set of linear equations:

(2) $$\rho \left(h\right)\;=\;\sum_{i=1}^{p}\;\alpha_{i}\;\rho \left(|h-i|\right)\;\mathrm{for}\;h=1,2,\ldots$$

These *Yule–Walker (YW) equations*, in turn, give rise to define the *partial* autocorrelation function (PACF), $\rho_{\mathrm{part}}\left(h\right)$ with time lags h∈N, in the following way (see Appendix A for further details): if $R_{k}:={\left(\rho (|i-j|)\right)}_{i,j=1,\ldots ,k}$ and $r_{k}:={\left(\rho (1),\ldots ,\rho (k)\right)}^{⊤}\in R^{k}$ for k=1,2,…, and if $a_{k}\in R^{k}$ denotes the solution of the equation $R_{k}\;a_{k}=r_{k}$, then the PACF at lag *k* is defined as the last component of $a_{k}$, i.e., $\rho_{\mathrm{part}}\left(k\right):=a_{k,k}$. Hence, if the YW-equations (2) hold, it follows that

(3) $$\rho_{\mathrm{part}}\left(p\right)\;=\;\alpha_{p},\;\rho_{\mathrm{part}}\left(h\right)\;=\;0\;\mathrm{for}\;\mathrm{all}\;h>p.$$

This characteristic abrupt drop-down of the PACF towards zero after lag h=p is commonly used for model identification in practice, namely by inspecting the sample PACF for such a pattern, see the Box–Jenkins program dating back to Box & Jenkins [3]. Details on the PACF’s computation are summarized in Appendix A. There, we also provide a brief discussion on some equivalences between ACF, PACF, and the AR-coefficients, in the sense that the AR(p) model (1) is characterized equivalently by either $\alpha_{1},\ldots ,\alpha_{p}$, or ρ(1),…,ρ(p), or $\rho_{\mathrm{part}}\left(1\right),\ldots ,\rho_{\mathrm{part}}\left(p\right)$.

Since the introduction of the ordinary AR(p) model, several other AR-type models have been proposed in the literature, not only for real-valued processes, but also for different types of quantitative processes such as count processes (and even for categorical processes), see the surveys by Holan et al. [4], Weiß [5]. In the present work, the focus is on (stationary and square-integrable) AR-type count processes ${\left(X_{t}\right)}_{Z}$, i.e., where the $X_{t}$ have a quantitative range contained in $N_{0}=\left\{0,1,\ldots \right\}$. Here, the AR(p) structure is implied by requiring the conditional mean at each time *t* to be linear in the last p observations [6], i.e.,

(4) $$E\left[X_{t}\;|\;X_{t-1},\ldots \right]\;=\;\alpha_{0}+\alpha_{1}\;X_{t-1}+\ldots +\alpha_{p}\;X_{t-p},$$

because then, the YW-equations (2) immediately follow by using the law of total covariance. Note that one also has to require $\alpha_{0}>0$ and $\alpha_{1},\ldots ,\alpha_{p}\geq 0$, as the counts $X_{t}$ are non-negative rvs having a truly positive mean, computed as $\mu =\alpha_{0}/\left(1-\alpha_{1}-\ldots -\alpha_{p}\right)$. The considered class of count processes satisfying (4) covers many popular special cases, such as the INAR(p) model (integer-valued AR) by Du & Li [7], the INARCH(p) model (‘CH’ = conditional heteroscedasticity) by Ferland et al. [8], or their bounded-counts counterparts discussed in Kim et al. [9]; see Section 2 for further details. These count processes satisfying (4), however, are not truly linear processes: by contrast to (1), there is no linear relation between their observations.

As all these AR(p)-like count processes satisfy the YW-equations (2) and, thus, the PACF characterization (3), it is common practice to employ the sample PACF (SPACF) for model identification given a count time series $X_{1},\ldots ,X_{n}$. More precisely, one commonly computes the SPACF from $X_{1},\ldots ,X_{n}$, ${\hat{\rho }}_{\mathrm{part}}\left(h\right)$ for h=1,2,…, and checks for the pattern (3) among those SPACF values that are classified as being significantly different from zero. An analogous procedure is common during a later step of the Box–Jenkins program. After having fitted a model to the data, one commonly computes the Pearson residuals to check the model adequacy; see Weiß [5], Jung & Tremayne [10] as well as Section 2. While, for an adequate model fit, the Pearson residuals are expected to be uncorrelated, significant SPACF values computed thereof would indicate that the fitted model does not adequately capture the true dependence structure. In both cases, practitioners usually evaluate the significance of ${\hat{\rho }}_{\mathrm{part}}\left(h\right)$ based on the following asymptotic result (see [11] (Theorem 8.1.2)):

(5) $$\sqrt{n}\;{\hat{\rho }}_{\mathrm{part}}\left(h\right)\;\overset{a}{∼}N\left(0,1\right)\;\mathrm{for}\;\mathrm{lags}\;h\geq p,$$

i.e., the value ${\hat{\rho }}_{\mathrm{part}}\left(h\right)$ is compared to the critical values $\pm z_{1-\alpha /2}/\sqrt{n}$ to test the null hypothesis of an AR(h−1) process on level α. Here, $N(\mu ,\sigma^{2})$ denotes the normal distribution with mean μ and variance $\sigma^{2}$, and $z_{\gamma }$ abbreviates the γ-quantile of N(0,1). The aforementioned critical values are automatically plotted in SPACF plots by common statistical software, e.g., if one uses the command pacf in R. However, Theorem 8.1.2 in Brockwell & Davis [11] assumes that the SPACF is computed from a truly linear AR(p) process as in (1), which is neither the case for the aforementioned AR-type count processes, nor for the Pearson residuals computed thereof. Thus, it is not clear if the approximation (5) is asymptotically correct and sufficiently precise in finite samples. In fact, some special asymptotic results in Kim & Weiß [12], Mills & Seneta [13], see Section 3 for further details, as well as some simulation results for Pearson residuals in Weiß et al. [14] indicate that this is generally indeed *not* the case.

Therefore, several alternative ways of implementing the PACF-test are presented in Section 4, namely relying on different types of bootstrap schemes for count time series. The performance of these bootstrap implementations compared to the asymptotic ones is analyzed in a comprehensive simulation study. In Section 5, we start with the case where the SPACF is applied to the original count time series $\left(X_{t}\right)$ with the aim of identifying the AR model order. Afterwards in Section 6, we consider the case of applying the SPACF to the (non-integer) Pearson residuals computed based on a model fit, i.e., the SPACF is used for checking the model adequacy. Our findings are also illustrated by a real-data example on claims counts in Section 7. Here, the computations and simulations in Section 5, Section 6 and Section 7 have been performed with the software R, and the documented R-code for Section 7 is provided in the Supplementary Materials to this article. Further R-codes can be obtained from the corresponding author upon request. We conclude the article in Section 8.

## 2. On AR-Type Count Time Series and Pearson Residuals

Several (stationary and square-integrable) AR-type count processes ${\left(X_{t}\right)}_{Z}$, which also have a conditional linear mean according to (4), have been discussed in the literature. Most of these processes either follow a model recursion using so-called thinning operators (typically referred to as INAR models), or they are defined by specifying the conditional distribution of $X_{t}|X_{t-1},\ldots$ together with condition (4), leading to INARCH models, see Weiß [5] for a survey. For this research, we focus on the most popular instance of these two classes, namely the INAR(p) model of Du & Li [7] on the one hand, and the INARCH(p) model of Ferland et al. [8] on the other hand.

The INAR(p) model of Du & Li [7] makes use of the binomial thinning operator “∘” introduced by Steutel & van Harn [15]. Having the parameter α∈(0;1) and being applied to a count rv *X*, it is defined by the conditional binomial distribution α∘X|X∼Bin(X,α), where the boundary cases are included as 0∘X=0 and 1∘X=X. Let ${\left(\epsilon_{t}\right)}_{Z}$ be square-integrable i. i. d. count rv, denote $\mu_{\epsilon }=E\left[\epsilon_{t}\right]$ and $\sigma_{\epsilon }^{2}=V\left[\epsilon_{t}\right]$. Then, the INAR(p) process ${\left(X_{t}\right)}_{Z}$ is defined by the recursion

(6) $$X_{t}\;=\;\alpha_{1}\circ X_{t-1}+\ldots +\alpha_{p}\circ X_{t-p}+\epsilon_{t},$$

where all thinnings are executed independently of each other, and where $\alpha_{•}:=\sum_{j=1}^{p}\alpha_{j}\;<1$ is assumed to ensure a stationary solution. The INAR(p) process (6) constitutes a pth-order Markov process, the transition probabilities of which are a convolution between the p binomial distributions $\mathrm{Bin}\left(X_{t-1},\alpha_{1}\right),\ldots ,\mathrm{Bin}\left(X_{t-p},\alpha_{p}\right)$ and the innovations’ distribution [16] (p. 469). The conditional mean satisfies (4) with $\alpha_{0}=\mu_{\epsilon }$, and the conditional variance is given by

(7) $$V\left[X_{t}\;|\;X_{t-1},\ldots \right]\;=\;\sigma_{\epsilon }^{2}+\sum_{j=1}^{p}\alpha_{j}\left(1-\alpha_{j}\right)\;X_{t-j},$$

see Drost et al. [16] (p. 469). The default choice for $\epsilon_{t}$ in the literature is a Poisson (Poi) distribution (which is the integer counterpart to the normal distribution), leading to the Poi-INAR(p) process. However, any other (non-degenerate) count distribution for $\epsilon_{t}$ might be used as well, such as the negative-binomial (NB) distribution for increased dispersion, leading to the NB-INAR(p) process. In the case of such a parametric specification for $\epsilon_{t}$, ones computes the moments $\mu_{\epsilon },\sigma_{\epsilon }^{2}$ according to this model, and then the conditional mean and variance according to (4) and (7), respectively.

The INARCH(p) model of Ferland et al. [8] directly assumes the conditional linear mean (4) to hold, and then specifies the conditional distribution of $X_{t}|X_{t-1},\ldots$ In Ferland et al. [8], the case of a conditional Poi-distribution is assumed, i.e., altogether

(8) $$X_{t}|X_{t-1},\ldots \;∼\;\mathrm{Poi}\left(\alpha_{0}+\alpha_{1}\;X_{t-1}+\ldots +\alpha_{p}\;X_{t-p}\right),$$

such that the conditional variance of this Poi-INARCH(p) process equals $V\left[X_{t}\;|\;X_{t-1},\ldots \right]=E\left[X_{t}\;|\;X_{t-1},\ldots \right]$. However, other choices for the conditional distribution of $X_{t}|X_{t-1},\ldots$ have been investigated in the literature; see [5] (Section 4.2).

For parameter estimation, one commonly uses either simple method-of-moment (MM) estimators (i.e., derived from marginal sample moments and the sample ACF, also see Appendix A), or the more advanced conditional maximum likelihood (CML) estimators, which are computed by using a numerical optimization routine (see [5] (Section 2.2)). It should be noted that for the INAR(p) model, also a semi-parametric specification exists (where the innovations’ distribution is left unspecified). The corresponding semi-parametric CML estimator was analyzed by Drost et al. [16]; see also the small-sample refinement by Faymonville et al. [17]. It leads to non-parametric estimates for the probabilities $p_{\epsilon ,k}=P\left(\epsilon_{t}=k\right)$ for *k* between some finite bounds 0≤l<u<∞ (and $p_{\epsilon ,k}=0$ for k∉{l,…,u}), which can then be used for computing $\mu_{\epsilon },\sigma_{\epsilon }^{2}$ as required for the conditional moments (4) and (7). More precisely, the *r*^th^ moment, r∈N, is given by $E\left[\epsilon_{t}^{r}\right]=\sum_{k=l}^{u}k^{r}\;p_{\epsilon ,k}$.

After having fitted a model to the count time series $X_{1},\ldots ,X_{n}$, a widely used approach for checking the model adequacy is to investigate the corresponding (standardized) Pearson residuals [5,10,14,18,19]. Let the parameters of the considered AR(p)-type model be comprised in the vector θ, and let $\hat{\theta }$ denote the estimated parameters of the fitted model. Furthermore, let us write the conditional mean as $E[X_{t}\;|\;X_{t-1},\ldots ;\;\theta ]$ and the conditional variance as $V[X_{t}\;|\;X_{t-1},\ldots ;\;\theta ]$ to express their dependence on the actual parameter values. Then, the Pearson residuals are defined as

(9) $$R_{t}:=R_{t}\left(\hat{\theta }\right)\;=\;\frac{X_{t}-E\left[X_{t}\;|\;X_{t-1},\ldots ;\;\hat{\theta }\right]}{\sqrt{V\left[X_{t}\;|\;X_{t-1},\ldots ;\;\hat{\theta }\right]}}\;\mathrm{for}\;t=p+1,\ldots ,n.$$

If the fitted AR(p)-type model is adequate for $X_{1},\ldots ,X_{n}$, then $R_{p+1},\ldots ,R_{n}$ should have a sample mean (variance) close to zero (one), and they should be uncorrelated. These necessary criteria are then used as adequacy checks. In the present research, our focus is on the SPACF computed from $R_{p+1},\ldots ,R_{n}$, which, for an adequate model fit, should not have values being significantly different from zero.

## 3. Some Asymptotic Results for the Sample PACF

The basic asymptotic result (5), which has been shown for the SPACF being computed from a true AR(p) process, has been extended in several directions. First, some refinements have been derived by Anderson [20,21] and further investigated by Kwan [22], who, however, assume the data-generating process (DGP) to be i. i. d. Gaussian, i.e., neither AR dependence nor count rvs are covered by their results. More precisely, Anderson [20] complements the asymptotic variance 1/n in (5) by the following O$\left(n^{-2}\right)$ approximation of the mean:

(10) $$E\left[{\hat{\rho }}_{\mathrm{part}}\left(h\right)\right]\;\overset{a}{=}\;\left\{\begin{array}{cc} -1/n\;+O(n^{-2}) & \mathrm{if}\;h\;\mathrm{odd},\\ -2/n\;+O(n^{-2}) & \mathrm{if}\;h\;\mathrm{even}. \end{array}\right.$$

While the Gaussian assumption is weakened by the statement that the result (10) “seems likely to have some validity for many non-Gaussian distributions” [20] (p. 406), the i. i. d.-assumption is not relaxed.

The O$\left(n^{-2}\right)$ approximation in (10) is extended to a corresponding O$\left(n^{-3}\right)$ approximation in Anderson [21] (pp. 565–566).

(11) $$\begin{array}{c} E\left[{\hat{\rho }}_{\mathrm{part}}\left(h\right)\right]\;\overset{a}{=}\;\left\{\begin{array}{cc} -\frac{1}{n}-\frac{h-1}{n^{2}}\;+O\left(n^{-3}\right) & \mathrm{if}\;h\;\mathrm{odd},\\ -\frac{2}{n}-\frac{h/2-2}{n^{2}}\;+O\left(n^{-3}\right) & \mathrm{if}\;h\;\mathrm{even}, \end{array}\right.\\ V\left[{\hat{\rho }}_{\mathrm{part}}\left(h\right)\right]\;\overset{a}{=}\;\frac{1}{n}-\frac{h+2}{n^{2}}\;+O\left(n^{-3}\right). \end{array}$$

While the O$\left(n^{-3}\right)$ extension in (11) seems relevant only for very small sample sizes *n*, the alternating pattern for the mean in (10) might affect the performance of the normal approximation also for larger *n*.

Another extension of the basic asymptotic result (5) is due to Kim & Weiß [12], Mills & Seneta [13]. These authors consider two particular types of AR(1) count process, namely a Poi-INAR(1) and a binomial AR(1) process, respectively, and derive an O$\left(n^{-2}\right)$ approximation of $V\left[{\hat{\rho }}_{\mathrm{part}}\left(h\right)\right]$ for h≥2. While their exact formulae are not relevant for the present research, the crucial point is as follows: In both cases, the approximate variance is of the form (1+c)/n, where *c* is inverse proportional to the mean μ, and also depends on the value of ρ(1). Especially for low means μ, the numerator 1+c deviates notably from 1. Hence, the basic asymptotics (5) do not hold for these types of count process. An analogous conclusion can be drawn from the simulation results in Weiß et al. [14] (Table 1), where the rejection rate for the SPACF of the Pearson residuals (with CML-fitted Poi-INAR(p) model) under the basic asymptotic critical values (5) is analyzed. These rejection rates are often below the intended level, which indicates that (5) does not hold here.

**Figure 1:**
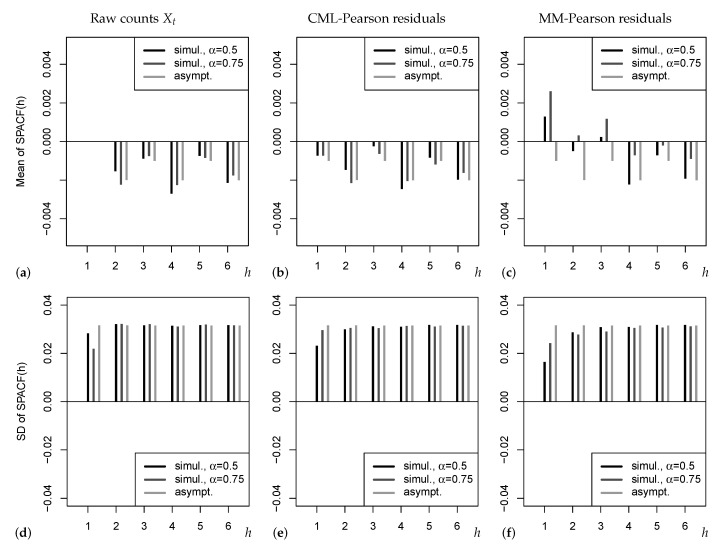
Means in (**a**–**c**) and SDs in (**d**–**f**) of SPACF(h) for sample size n=1000, either simulated values for Poi-INAR(1) DGP with μ=5 and AR-parameter α, or asymptotic values from (11). SPACF computed from raw counts, and from Pearson residuals with CML or MM estimation.

These possible drawbacks of existing asymptotic results are illustrated by Figure 1. The upper panel refers to the mean of SPACF(h), which is either computed from $10^{4}$ simulated Poi-INAR(1) time series (black and dark grey bars), or according to the refined asymptotic result (11) (light grey bars). Note that the sample size n=1000 was chosen rather large such that sample properties and (true) asymptotic properties should agree reasonably well. In Figure 1a, where the SPACF is computed from the raw counts $\left(X_{t}\right)$, we omit plotting the mean at h=1 as this would violate the graphic’s Y-range (recall that $\rho_{\mathrm{part}}\left(1\right)=\alpha$). From (a) and (b), we recognize that the simple asymptotics (5), where the mean of SPACF is approximated by zero, would be misleading in practice, because a negative bias with an oscillating pattern (odd vs. even lags) is observed. As a consequence, if testing the PACF based on (5) and thus ignoring the bias, we may get unreliable sizes, which is also later observed in our simulation studies. The alternating pattern of the bias in (a) and (b) is similar to the refined asymptotics (11). However, we do not observe an exact agreement to (11), as the simulated means seem to depend on the actual value of the AR-parameter α. The effect of α gets much stronger in (c), where even positive bias values for low *h* are observed, contradicting (11). This is caused by the use of the MM estimator, which is known to be increasingly biased with increasing α[23]; a possible solution for practice could be to use a bias-corrected version of the MM estimator. The lower panel in Figure 1 shows the corresponding standard deviations (SDs). The strongest deviation between simulated and asymptotic results is observed for lag h=1, followed by lag h=2. In particular, for both types of Pearson residuals and both h=1,2, the asymptotic SD from (11) is too large (and the asymptotic SD according to (5) would even be larger) such that a corresponding PACF-test is expected to be conservative (which is later confirmed by our simulation study). Therefore, it seems advisable to look for other ways of implementing the PACF-test, neither relying on (5) nor (11). An approximation based on asymptotic results does not look promising in general, as we expect the asymptotics to highly depend on the actual DGP, recall the aforementioned results by Kim & Weiß [12], Mills & Seneta [13]. Thus, in what follows, our idea is to try out different types of bootstrap implementations, i.e., the true distribution of the SPACF is approximated by appropriate resampling schemes. This might also allow to account for the effect of the selected estimator when computing the Pearson residuals.

## 4. Bootstrap Approaches for the Sample PACF

Let ϑ denote the parameter of interest for the actual DGP $\left(Y_{t}\right)$, and let $\hat{\vartheta }=T\left(Y_{1},\ldots ,Y_{n}\right)$ denote an estimate of it (in the present research, this parameter is the (S)PACF at some lag h∈N). Analogously, let $\left(Y_{t}^{*}\right)$ denote a corresponding bootstrap DGP, and let ${\hat{\vartheta }}^{*}=T\left(Y_{1}^{*},\ldots ,Y_{n}^{*}\right)$ be the estimator obtained from a bootstrap sample. If $E^{*}\left[\cdot \right]$ denotes the expectation operator of the bootstrap DGP, that is, conditional on the data $X_{1},\ldots ,X_{n}$, then the centered bootstrap estimate is given by ${\hat{\vartheta }}_{cent}^{*}:={\hat{\vartheta }}^{*}-E^{*}\left[{\hat{\vartheta }}^{*}\right]$. A common approach for constructing a two-sided bootstrap confidence interval (CI) for ϑ with confidence level 1−α∈(0;1) is given by

(12) $$\left[\hat{\vartheta }-q_{1-\alpha /2}\left({\hat{\vartheta }}_{\mathrm{cent}}^{*}\right);\;\hat{\vartheta }-q_{\alpha /2}\left({\hat{\vartheta }}_{\mathrm{cent}}^{*}\right)\right],$$

where $q_{\gamma }\left(\cdot \right)$ denotes the γ-quantile, see Hall [24]. The bootstrap CI (12) is used for testing the null hypothesis “$H_{0}:\vartheta =\vartheta_{0}$” on level α by applying the following decision rule: reject $H_{0}$ if $\vartheta_{0}$ is not contained in the CI (12). This implies the equivalent decision rule to reject $H_{0}$ if

(13) $$\hat{\vartheta }-\vartheta_{0}<q_{\alpha /2}\left({\hat{\vartheta }}_{\mathrm{cent}}^{*}\right)\;\mathrm{or}\;\hat{\vartheta }-\vartheta_{0}>q_{1-\alpha /2}\left({\hat{\vartheta }}_{\mathrm{cent}}^{*}\right).$$

In the present article, ϑ refers to the PACF at lag *h*, computed from either the original count process $\left(X_{t}\right)$, or from the Pearson residuals $\left(R_{t}\right)$ obtained after model fitting. In both cases, the PACF at lag *h* is tested against the hypothetical value $\vartheta_{0}=0$, as it would be the case for an AR-type process of order <h.

If we apply the PACF to the original count time series $X_{1},\ldots ,X_{n}$, then the following setups are considered:

- fully parametric setup: a fully parametric count AR(p) model with p≤2 is fitted to the data and then used as the bootstrap DGP; the PACF at certain lags h>p is tested against zero. Here, we focus on the Poi-INAR(p) model, and we use the parametric INAR-bootstrap of Jentsch & Weiß [25].
- semi-parametric setup: a semi-parametric count AR(p) model is fitted to the data [16] and then used as the bootstrap DGP; the PACF at lags h>p is tested against zero. Here, we focus on the INAR(p) model with unspecified innovations, and we use the semi-parametric INAR-bootstrap of Jentsch & Weiß [25].
- non-parametric setup: we use the circular block bootstrap as considered by Politis & White [26], where an automatic block-length selection might be done by using the function b.star in R-package “np” (https://CRAN.R-project.org/package=np, accessed on 31 March 2022).

In case of an INAR(p) bootstrap DGP, the centering at lag *h* is done by the lag-*h* PACF corresponding to the fitted model, i.e., which satisfies the YW-equations (2) under estimated parameters, see Appendix A for computational details. In case of the non-parametric block bootstrap, the sample PACF at lag *h* is used for centering the bootstrap values.

If we apply the PACF to the Pearson residuals $\left(R_{t}\right)$, then again (semi-)parametric setups are considered, where also model fitting is replicated based on the bootstrap time series, as well as the subsequent computation of Pearson residuals based on the bootstrap model fit. This time, a centering is not necessary. Non-parametric bootstrap schemes can be directly applied to the original Pearson residuals (without the need for model fitting during bootstrap replication). Under the null of model adequacy, we expect the available Pearson residuals to be uncorrelated. Thus, a first idea is to apply the classical Efron bootstrap [27], although this bootstrap scheme actually requires i. i. d. data. Therefore, as a second idea, we also apply the aforementioned block bootstrap to $\left(R_{t}\right)$ to account for possible non-linear dependencies.

**Remark** **1.** *For implementing the (semi-)parametric INAR bootstraps, or for computing the Pearson residuals with respect to an INAR model, the model parameters have to be estimated. The following approaches are used for this purpose:*

- *If the fully parametric Poi-INAR(p) model is fitted, we use either the MM estimator of $\theta =(\alpha_{1},\ldots ,\alpha_{p},\mu_{\epsilon })$, which is obtained by solving the mean equation $\mu =\mu_{\epsilon }/\left(1-\alpha_{1}-\ldots -\alpha_{p}\right)$ as well as the YW-equations *(2)* for h=1,…,p in $\mu_{\epsilon },\alpha_{1},\ldots ,\alpha_{p}$ and by plugging-in the sample counterparts for μ,ρ(1),…,ρ(p), or the CML estimator of θ. The latter is obtained by numerically maximizing the conditional log-likelihood function $\ell \left(\theta \;|\;x_{p},\ldots ,x_{1}\right)\;=\;\sum_{t=p+1}^{T}\ln p(x_{t}\;|\;x_{t-1},\ldots ,x_{t-p},\;\theta )$, where the transition probabilities $p(x_{t}\;|\;x_{t-1},\ldots ,x_{t-p})$ are computed by evaluating the convolution of the *p* thinnings’ binomial distributions and the innovations’ Poisson distribution, i.e., $\mathrm{Bin}\left(x_{t-1},\alpha_{1}\right)★\ldots ★\mathrm{Bin}\left(x_{t-p},\alpha_{p}\right)★\mathrm{Poi}\left(\mu_{\epsilon }\right)$.*
- *If the semi-parametric Poi-INAR(p) model is fitted, then the innovations’ distribution is not specified. As a result, the parameter vector now equals $\theta_{sp}=\left(\alpha_{1},\ldots ,\alpha_{p},p_{\epsilon ,0},p_{\epsilon ,1},\ldots \right)$, and we use the semi-parametric CML approach of Drost et al. [16] for estimation. In this case, the transition probabilities for the log-likelihood function $\ell (\theta_{sp}\;|\;x_{p},\ldots ,x_{1})$ are obtained from the convolution $\mathrm{Bin}\left(x_{t-1},\alpha_{1}\right)★\ldots ★\mathrm{Bin}\left(x_{t-p},\alpha_{p}\right)★G_{\epsilon }$, where $G_{\epsilon }$ expresses the unspecified innovations’ distribution with probability masses $p_{\epsilon ,0},p_{\epsilon ,1},\ldots$*

## 5. PACF Diagnostics for Raw Counts

In the first part of our simulation study, we analyze the performance of the asymptotic and (semi-)parametric implementations of PACF-tests if these are applied to the raw counts $\left(X_{t}\right)$ (the results of the non-parametric bootstrap schemes are discussed separately in Remark 2). We consider 1st- and 2nd-order AR-type DGPs, where the aim of applying the PACF-tests (nominal level 0.05) is the identification of the correct AR-order p. As the bootstrap versions of these tests are computationally very demanding (especially the semi-parametric INAR bootstrap), we use the warp-speed method of Giacomini et al. [28] for executing the simulations. This, in turn, allows us to use $10^{4}$ replications throughout our simulation study. We also cross-checked that the achieved rejection rates are close to those obtained by a traditional bootstrap implementation with B=500 bootstrap replications per simulation run. All simulations have been done with the software R, and R-codes can be obtained from the corresponding author upon request.

Table 1 shows the rejection rates of the PACF-tests for different types of AR(1)-like count DGP, recall Section 2. There, the PACFs are computed from a simulated count time series $x_{1},\ldots ,x_{n}$ of length *n*, where the choice n=100 (n=1000) represents the small (large) sample behaviour. The results refer to the medium autocorrelation case ρ(1)=0.5, but further results for ρ(1)∈{0.25,0.75} are summarized in Appendix B, see Table A1. Five implementations of the PACF-test are considered: using the simple asymptotic approximation (5) or the refined one (11) (recall Section 3), using the parametric Poi-INAR(1) bootstrap with either MM or CML estimates, and using the semi-parametric INAR(1) bootstrap with CML estimates (recall Section 4). If first looking at the block “Poi-INAR(1) DGP” in Table 1, we recognize that all implementations perform roughly the same, i.e., the rejection rate at lag h=1 (expressing the power of the PACF-test) is close to 1, and the rejection rates at lags h≥2 (expressing the size) are close to the 0.05-level. It should be noted, however, that for ρ(1)=0.25, see Table A1, the asymptotic implementations have notably less power at lag h=1. An analogous conclusion holds for the NB-INAR(1) block in Table 1, although now, the model behind the parametric Poi-INAR(1) bootstrap is misspecified. So the parametric bootstrap exhibits robustness properties in finite samples. In the third block, “Poi-INARCH(1)”, also the semi-parametric bootstrap is misspecified, but again the rejection rates are robust for ρ(1)=0.5. For ρ(1)=0.75 in Table A1, however, we observe size exceedences for lags h≥2, i.e., the misspecification of Poi-INARCH(1) as Poi-INAR(1) is not negligible anymore for this DGP. This is plausible in view of Remark 4.1.7 in Weiß [5], where it is shown that these models lead to different sample paths for high autocorrelation. Much more surprising, also both asymptotic implementations deteriorate (even more severely) for a Poi-INARCH(1) DGP with ρ(1)=0.75, see Table A1, i.e., we get too many false rejections in any case. Thus, if one anticipates that the data are generated by an INARCH process, a tailor-made parametric bootstrap implementation of the PACF-tests should be used.

Let us continue our performance analyses by turning to 2nd-order DGPs. In Table 2, the (semi-)parametric bootstrap schemes are still executed by (erroneously) assuming a 1st-order INAR DGP (like in Table 1), i.e., they are affected by a (further) source of model misspecification. But as seen from the rejection rates in Table 2, we still have good size (h≥3) and power values (h=1,2), comparable to those of the refined asymptotic implementation (11). By contrast, the simple asymptotic (5) leads to a clearly reduced power at lag h=2. Finally, in Table 3, the bootstrap schemes now correctly assume a 2nd-order INAR DGP, i.e., we only have the following misspecifications left: parametric Poi-INAR(2) bootstrap applied to NB-INAR(2) or Poi-INARCH(2) DGP, and semi-parametric INAR(2) bootstrap applied to Poi-INARCH(2) DGP. It can be seen that the parametric bootstrap using MM estimates as well as the semi-parametric bootstrap lead to improved power at lag h=2, whereas the parametric CML-setup even deteriorates (especially under Poi-INARCH(2) misspecification). The latter observation fits well to later results in Section 6, where the parametric bootstrap with CML estimates does again worse than its MM- or semi-CML-counterparts. This can be explained by the fact that for a fully parametric CML approach, model misspecification affects the estimates of all parameters simultaneously, while for the MM approach, for example, the estimation of mean and dependence parameters coincide across all three types of DGP. So it does not seem advisable to use a fully parametric bootstrap in combination with CML estimation for PACF diagnostics.

To sum up, if computing the SPACF from the raw counts $\left(X_{t}\right)$, with the aim of identifying the AR-order of the given count DGP, the overally best performance is shown by the MM-based parametric and CML-based semi-parametric bootstrap implementation of the PACF-test, but also the refined asymptotic implementation relying on (11) does reasonably well. The latter is remarkable as these asymptotics are not the correct ones regarding the considered count DGPs (also recall the discussion of Figure 1), but it appears that their approximation quality is sufficient anyway. The simple asymptotic implementation (5), by contrast, as it is used by statistical software packages by default, leads to reduced power in some cases. From a practical point of view, as the additional benefit of the (semi-)parametric bootstrap schemes compared to the refined asymptotic implementation (11) is not that large, especially in view of the necessary computational effort, it seems advisable for practice to use (11) for doing the PACF-test. Recall that this recommendation refers to the case, where the SPACF is computed from the raw counts $\left(X_{t}\right)$ to identify the DGP’s AR-order. The case of applying the PACF-test to Pearson residuals for checking the model adequacy is analyzed in the following Section 6.

## 6. PACF Diagnostics for Pearson Residuals

While the raw counts’ SPACF is typically computed before model fitting (namely for identifying appropriate candidate models), the PACF-analysis of the Pearson residuals is relevant after model fitting, namely for checking the fitted model’s adequacy. Thus, the main difference of the simulations in the present section, compared to those of Section 5, is given by the fact that this time, we first fit a (Poi-)INAR model to the data, and then we apply the SPACF to the Pearson residuals computed thereof. For Poi-INAR model fitting, we again use either MM- or CML-estimation, and then we apply the asymptotic or corresponding parametric bootstrap implementations (like before, we use the warp-speed method). An exception is given by the semi-parametric CML estimation, as in this case, also the semi-parametric bootstrap is used for methodological consistency (and Pearson residuals are computed with respect to an unspecified INAR model). We also consider the same scenarios of model orders as before, i.e., 1st-order DGPs and INAR(1)-fit (Table 4 and Table 7), 2nd-order DGPs but still INAR(1)-fit (Table 5 and Table 8), and 2nd-order DGPs and INAR(2)-fit (Table 6 and Table 9). Recall that the fitted model is now used for both the computation of the Pearson residuals and the implementation of (semi-)parametric bootstrap schemes.

**Table 4:** Rejection rates of PACF-tests applied to Pearson residuals using MM estimates (DGPs with μ=5 and ρ(1)=0.5), where both residuals and parametric bootstrap rely on null of Poi-INAR(1) process.

True DGP:		Poi-INAR(1)				NB-INAR(1), $\frac{\sigma^{2}}{\mu }=1.5$				Poi-INARCH(1)			
		PACF at Lag h=				PACF at Lag h=				PACF at Lag h=			
**Method**	n	**1**	**2**	**3**	**4**	**1**	**2**	**3**	**4**	**1**	**2**	**3**	**4**
asym.	100	0.000	0.026	0.040	0.043	0.001	0.026	0.038	0.044	0.000	0.031	0.041	0.044
(5)	1000	0.000	0.029	0.044	0.053	0.000	0.030	0.043	0.052	0.001	0.033	0.044	0.050
asym.	100	0.001	0.030	0.046	0.050	0.001	0.032	0.042	0.049	0.001	0.037	0.049	0.049
(11)	1000	0.000	0.030	0.047	0.048	0.000	0.030	0.047	0.051	0.000	0.032	0.047	0.050
param.	100	0.056	0.051	0.052	0.048	0.050	0.049	0.049	0.045	0.066	0.050	0.047	0.051
MM	1000	0.051	0.053	0.049	0.045	0.050	0.051	0.053	0.051	0.060	0.046	0.050	0.044

Let us start with the case of fitting a Poi-INAR model by MM estimation, see Table 4, Table 5 and Table 6. In Table 4 (1st-order models and DGPs; also see Table A3 in the Appendix B), we recognize that both asymptotic implementations lead to undersizing at lags h=1,2 (particularly severe at h=1). This is in close agreement to our conclusions drawn from Figure 1 as well as to the findings of Weiß et al. [14]. An analogous observation can be done in Table 6 (2nd-order models and DGPs), but now for lags h=1,2,3 (particularly severe at h=1,2). In both cases, however, the MM-based parametric bootstrap holds the nominal 0.05-level reasonably well. The drawback resulting from this undersizing gets clear in Table 5, where the wrong AR-order was selected during model fitting: the asymptotic implementations lead to a very low power for sample size n=100, implying that one will hardly recognize the inadequate model choice. Thus, if model assumptions are used anyway for computing the Pearson residuals, the asymptotic implementation should be avoided, but the model assumptions should also be utilized for executing the PACF-test by using the parametric bootstrap scheme. As a final remark, strictly speaking, we are always concerned with model misspecification if having an NB-INAR or Poi-INARCH DGP. However, all three DGPs per table have the same conditional mean and, thus, the same autocorrelation structure, only their conditional variances differ. Also the MM-estimates required for computing the conditional mean are identical across all models. Thus, it is not surprising that the rejection rates of the PACF-tests do not differ much among these three types of DGP (but again with slight oversizing for the Poi-INARCH DGPs).

Finally, we did the same simulations again, but using CML instead of MM estimation. Table 7 (as well as Table A5 in the Appendix B) refer to the case of both 1st-order models and 1st-order DGPs. In the first block, where the parametric Pearson residuals are computed by correctly assuming a Poi-INAR(1) DGP, we have again strong undersizing at lag 1 for the asymptotic implementation, but a close agreement to the nominal 0.05-level for the parametric bootstrap. The remaining blocks with NB-INAR(1) and Poi-INARCH(1) DGP, however, differ notably from the corresponding blocks in Table 7 and Table A5, respectively. This is plausible as the parametric CML approach for a misspecified model leads to misleading estimates for all parameters. While MM estimation leads to the same estimates for the dependence parameters across the three 1st-order models, these differ for parametric CML estimation. Therefore, we have high rejection rates especially at lag 1 (especially if using the parametric bootstrap), which is desirable on the one hand as the fitted model is indeed not adequate. On the other hand, we did not misspecify the (P)ACF structure (a 1st-order model is correct for all DGPs) but the actual data-generating mechanism, i.e., a user might draw the wrong conclusion from this rejection based on the lag-1 PACF. At this point, it is interesting to look at the semi-parametric model fit and bootstrap in Table 7. For both INAR(1) DGPs, the rejection rates are close to the 0.05-level, which is the desirable result as we are concerned with an adequate model fit. For the Poi-INARCH(1) DGP, by contrast, we get moderately increased rejection rates at lag 1, which again has to be assessed ambivalently: on the one hand, the fitted INAR(1) model is indeed not adequate, but on the other hand, the inadequacy does not refer to the autocorrelation structure.

**Table 5:** Like Table 4, but 2nd-order DGPs with $\alpha_{2}=0.2$.

True DGP:		Poi-INAR(2)				NB-INAR(2), $\frac{\sigma^{2}}{\mu }=1.5$				Poi-INARCH(2)			
		PACF at Lag h=				PACF at Lag h=				PACF at Lag h=			
**Method**	n	**1**	**2**	**3**	**4**	**1**	**2**	**3**	**4**	**1**	**2**	**3**	**4**
asym.	100	0.016	0.264	0.079	0.043	0.014	0.266	0.077	0.043	0.018	0.275	0.080	0.045
(5)	1000	0.975	0.998	0.648	0.191	0.958	0.998	0.625	0.192	0.966	0.999	0.642	0.191
asym.	100	0.013	0.359	0.104	0.061	0.010	0.356	0.100	0.059	0.014	0.369	0.105	0.060
(11)	1000	0.972	0.998	0.662	0.212	0.954	0.998	0.640	0.210	0.962	0.999	0.655	0.210
param.	100	0.395	0.365	0.102	0.063	0.369	0.356	0.094	0.064	0.396	0.378	0.092	0.069
MM	1000	1.000	0.999	0.664	0.201	1.000	0.999	0.646	0.204	1.000	0.999	0.662	0.211

**Table 6:** Rejection rates of PACF-tests applied to Pearson residuals using MM estimates (DGPs with μ=5, ρ(1)=0.5, and $\alpha_{2}=0.2$), where both residuals and parametric bootstrap rely on null of Poi-INAR(2) process.

True DGP:		Poi-INAR(2)				NB-INAR(2), $\frac{\sigma^{2}}{\mu }=1.5$				Poi-INARCH(2)			
		PACF at Lag h=				PACF at Lag h=				PACF at Lag h=			
**Method**	n	**1**	**2**	**3**	**4**	**1**	**2**	**3**	**4**	**1**	**2**	**3**	**4**
asym.	100	0.000	0.001	0.033	0.034	0.000	0.002	0.035	0.038	0.000	0.002	0.034	0.036
(5)	1000	0.000	0.001	0.036	0.042	0.000	0.001	0.033	0.042	0.000	0.001	0.036	0.040
asym.	100	0.000	0.003	0.041	0.043	0.000	0.003	0.040	0.044	0.000	0.002	0.042	0.046
(11)	1000	0.000	0.001	0.036	0.042	0.000	0.001	0.035	0.044	0.000	0.001	0.036	0.045
param.	100	0.050	0.037	0.044	0.048	0.053	0.038	0.047	0.050	0.052	0.038	0.059	0.050
MM	1000	0.049	0.049	0.046	0.052	0.059	0.049	0.052	0.052	0.067	0.054	0.055	0.053

**Table 7:** Rejection rates of PACF-tests applied to Pearson residuals using CML estimates (DGPs with μ=5 and ρ(1)=0.5), where both residuals and bootstrap rely on null of Poi-INAR(1) process (parametric bootstrap) or unspecified INAR(1) process (semi-parametric bootstrap), respectively.

True DGP:		Poi-INAR(1)				NB-INAR(1), $\frac{\sigma^{2}}{\mu }=1.5$				Poi-INARCH(1)			
		PACF at Lag h=				PACF at Lag h=				PACF at Lag h=			
**Method**	n	**1**	**2**	**3**	**4**	**1**	**2**	**3**	**4**	**1**	**2**	**3**	**4**
asym.	100	0.009	0.035	0.041	0.046	0.028	0.036	0.041	0.039	0.023	0.038	0.045	0.042
(5)	1000	0.008	0.035	0.045	0.048	0.902	0.182	0.069	0.053	0.745	0.148	0.072	0.051
asym.	100	0.009	0.041	0.046	0.048	0.043	0.055	0.047	0.050	0.034	0.053	0.048	0.051
(11)	1000	0.009	0.039	0.046	0.053	0.909	0.198	0.077	0.058	0.753	0.167	0.072	0.055
param.	100	0.052	0.049	0.049	0.045	0.238	0.062	0.048	0.049	0.209	0.064	0.053	0.050
CML	1000	0.049	0.053	0.049	0.053	0.993	0.226	0.075	0.051	0.963	0.188	0.082	0.048
semi-p.	100	0.050	0.051	0.054	0.053	0.057	0.048	0.052	0.044	0.070	0.052	0.049	0.051
CML	1000	0.039	0.053	0.056	0.048	0.052	0.053	0.055	0.049	0.225	0.067	0.058	0.050

**Table 8:** Like Table 7, but 2nd-order DGPs with $\alpha_{2}=0.2$.

True DGP:		Poi-INAR(2)				NB-INAR(2), $\frac{\sigma^{2}}{\mu }=1.5$				Poi-INARCH(2)			
		PACF at Lag h=				PACF at Lag h=				PACF at Lag h=			
**Method**	n	**1**	**2**	**3**	**4**	**1**	**2**	**3**	**4**	**1**	**2**	**3**	**4**
asym.	100	0.026	0.301	0.084	0.043	0.001	0.403	0.090	0.044	0.001	0.404	0.092	0.045
(5)	1000	0.522	0.999	0.696	0.192	0.001	1.000	0.718	0.178	0.000	1.000	0.726	0.174
asym.	100	0.020	0.391	0.110	0.061	0.002	0.502	0.114	0.059	0.001	0.492	0.125	0.064
(11)	1000	0.508	0.999	0.709	0.212	0.001	1.000	0.733	0.193	0.000	1.000	0.727	0.193
param.	100	0.099	0.399	0.114	0.059	0.031	0.514	0.105	0.062	0.028	0.495	0.131	0.063
CML	1000	0.840	1.000	0.710	0.235	0.041	1.000	0.755	0.183	0.023	1.000	0.737	0.194
semi-p.	100	0.222	0.350	0.106	0.063	0.172	0.384	0.098	0.054	0.134	0.410	0.109	0.066
CML	1000	0.999	0.999	0.664	0.222	0.976	0.999	0.665	0.204	0.917	1.000	0.720	0.208

**Table 9:** Rejection rates of PACF-tests applied to Pearson residuals using CML estimates (DGPs with μ=5, ρ(1)=0.5, and $\alpha_{2}=0.2$), where both residuals and bootstrap rely on null of Poi-INAR(2) process (parametric bootstrap) or unspecified INAR(2) process (semi-parametric bootstrap), respectively.

True DGP:		Poi-INAR(2)				NB-INAR(2), $\frac{\sigma^{2}}{\mu }=1.5$				Poi-INARCH(2)			
		PACF at Lag h=				PACF at Lag h=				PACF at Lag h=			
**Method**	n	**1**	**2**	**3**	**4**	**1**	**2**	**3**	**4**	**1**	**2**	**3**	**4**
asym.	100	0.002	0.002	0.033	0.036	0.003	0.001	0.037	0.038	0.001	0.001	0.037	0.036
(5)	1000	0.000	0.000	0.037	0.044	0.303	0.021	0.082	0.063	0.158	0.005	0.063	0.047
asym.	100	0.002	0.002	0.039	0.041	0.005	0.004	0.047	0.049	0.002	0.002	0.042	0.046
(11)	1000	0.000	0.001	0.038	0.044	0.332	0.028	0.089	0.070	0.174	0.006	0.074	0.055
param.	100	0.002	0.002	0.037	0.043	0.003	0.003	0.051	0.047	0.002	0.002	0.048	0.051
CML	1000	0.000	0.001	0.038	0.044	0.344	0.023	0.086	0.067	0.186	0.007	0.065	0.058
semi-p.	100	0.043	0.038	0.050	0.044	0.044	0.036	0.047	0.043	0.038	0.039	0.052	0.050
CML	1000	0.035	0.045	0.053	0.053	0.051	0.054	0.055	0.051	0.149	0.056	0.054	0.053

Essentially analogous conclusions can be drawn from Table 9, where we are concerned with both 2nd-order models and 2nd-order DGPs. So let us turn to Table 8, where 1st-order models are fitted to 2nd-order DGPs. Thus, we are concerned with at least an inadequate autocorrelation structure (and sometimes also further model misspecification) such that high rejection rates are desirable. Let us start with the first block about the Poi-INAR(2) DGP. As a consequence of the strong undersizing at lag 1, the parametric bootstrap, and especially the asymptotic implementations, show relatively low power values, especially for the small sample size n=100. The semi-parametric bootstrap, by contrast, has substantially higer power values at lag 1. For lags h≥2, the rejection rates are similar between the different implementations, with a slight advantage for the refined asymptotics as well as the parametric bootstrap. The discrepancy at lag 1 gets even more extreme for the NB-INAR(2) and Poi-INARCH(2) DGP, then all other implementations than the semi-parametric one lead to power close to zero. For lags 2 and 3, by contrast, the refined asymptotics as well as the parametric bootstrap are again more powerful. However, looking back to Table 5, it seems that the overall most appealing power is shown by the MM-based parametric bootstrap. This type of bootstrap also has the advantage that the necessary computational effort is much less than for the CML-based bootstraps. Thus, altogether, while we recommended to use the refined asymptotics (11) if testing the PACF computed from the raw counts, the PACF analysis of Pearson residuals should be done by the MM-based parametric bootstrap: if computing the Pearson residuals from an MM-fitted Poi-INAR model, and if using this model fit for parametric bootstrap, one has good size properties and an appealing power performance at the same time. Certainly, this recommendation does not exclude to do CML-fitting in a second step, once the correct AR-order has been identified. But during the phase of model diagnostics, at least if *n* is not particularly large, the parametric-MM solution seems to be best suited.

**Remark** **2.** *As mentioned in Section 4, we also tried out fully non-parametric bootstrap schemes. For the case where the PACF-tests are applied to the raw counts $\left(X_{t}\right)$, as discussed in Section 5, the circular block bootstrap was used as a fully non-parametric setup, see Table A2 in the Appendix B for the obtained results. While these implementations lead to an appealing power at lag h=1, strong size deteriorations are observed for h≥2. The strongest deviations are observed for the fixed block length b=5. Increasing b, first the low-lag rejection rates stabilize at 0.05, while we have undersizing for large lags. For b=20,25, we have good sizes for h=5,6, but now the low lags lead to exceedances of 0.05. Thus, tailor-made block lengths would be required for different lags h. The automatic block-length selection via* *b.star* *typically leads to block lengths between 5 and 10 (depending on the actual extent of ρ(1)), but this causes undersizing throughout, getting more severe for increasing h. The reason why* *b.star* *tends to pick block lengths that are too small to capture dependence at larger lags is given by the fact that it is designed to select a block length suitable for inference about the sample mean, but not for the sample PACF. In view of the aforementioned size problems and the unclear choice of block lengths, we discourage from using block-bootstrap implementations of the PACF-test for analyzing the raw counts data.*

*If doing a PACF-analysis of the Pearson residuals, as we investigate it in the present Section 6, then, besides block-bootstrap implementations, also the Efron bootstrap appears reasonable for this task. For the case where the Pearson residuals rely on MM estimates, simulation results are summarized in Table A4 in the Appendix B. If doing an automatic block-length selection via* *b.star* *, we often end up with block length 1 (as the Pearson residuals are uncorrelated under model adequacy). Thus, the* *b.star* *-block bootstrap shows nearly the same rejection rates as the Efron bootstrap, but these are too low at lags h=1,2, like for the asymptotic implementations. Increasing the block length to the fixed values b=5 or b=10, we get an even further decrease in size. Therefore, neither Efron nor block bootstrap offer any advantage compared to the asymptotic implementations. Analogous conclusions hold if model fitting is done by CML estimation, see Table A6 in the Appendix B, so we discourage from the use of Efron and block bootstrap also if doing a PACF-test of the Pearson residuals.*

## 7. Real-Data Application

**Figure 2:**
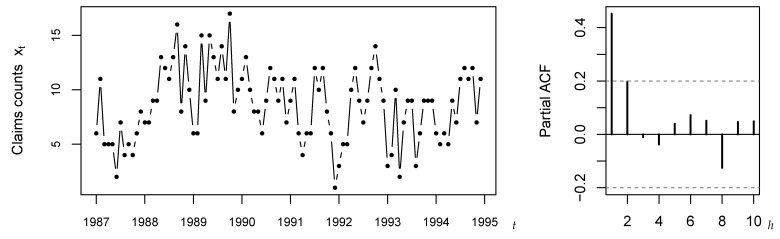
Time series plot and SPACF(h) of claims counts, see Section 7.

For illustration, we pick up a widely discussed data example from the literature, namely the claims counts data introduced by Freeland [29]. These counts express the monthly number of claims caused by burn-related injuries in the heavy manufacturing industry for the period 1987–1994, i.e., the count time series is of length n=96; see Figure 2. Recall that the R-code used for the subsequent computations is provided in the Supplementary Materials. Freeland [29] suggested to model these data by a Poi-INAR(1) model, but following the discussions of subsequent authors, this model choice is not without controversy. For example, the marginal distribution exhibits moderate overdispersion, as the sample variance 11.357 exceeds the mean 8.604. Therefore, some authors suggested to consider an NB-INAR(1) or Poi-INARCH(1) model instead. Furthermore, one may doubt the 1st-order AR-structure, see Weiß et al. [30], as the SPACF in Figure 2 is only slightly non-significant at lag h=2, where the plotted critical values (dashed lines) refer to the PACF-test on level 0.05 based on the simple asymptotic implementation (5). Thus, altogether, we are concerned with a scenario that fits very well to our simulation study in Section 5 and Section 6: the null hypothesis for the data is that of a Poi-INAR(1) model, but this model might be misspecified in terms of marginal distribution, model order, or the actual AR-type data-generating mechanism. Moreover, the sample size n=96 and the lag-1 sample ACF 0.452 are close to the parametrizations considered there. In what follows, we apply the different implementations of the PACF-test to (the Pearson residuals computed from) the claims data. Certainly, as we do not know the true model behind the data, we are not in a position to pass definitive judgement on whether or not a test lead to the correct or wrong decision. But we shall discuss the PACF-tests with respect to our simulation results.

Let us start with an analysis of the raw counts’ SPACF, in analogy to Section 5. Table 10 summarizes the SPACF(h) values for h=1,…,5 (bold font) as well as the corresponding critical values (level 0.05). The latter are computed by the five methods considered in Section 5, with the number of bootstrap replications chosen as B=1000. For the simple asymptotic implementation (5), as we have already seen in Figure 2, we get a rejection only at lag 1, whereas the remaining methods reject also at lag 2. Thus, there is indeed evidence that the data might stem from a higher-order model. In addition, the different lag-2 decisions for (5) vs. the remaining implementations appear plausible in view of Table 2, where we found clearly lower power for (5) at h=2. Note that all critical values except (5) are visibly asymmetric, so the SPACF appears rather biased for n=96. Furthermore, all bootstrap implementations lead to quite similar critical values, and the refined asymptotic implementation (11) is also similar to them except for the upper critical value at h=1.

Next, we fit either a Poi-INAR(1) model to the claims counts (via MM or CML), or an unspecified INAR(1) model by the semi-parametric CML approach. Using the resulting model fits, we first compute a set of Pearson residuals for each model, and then the SPACF thereof, like in Section 6. The critical values are determined by both asymptotic approaches as well as by the bootstrap approach corresponding to the respective estimation method. Results are summarized in Table 11. We get only a few rejections anymore, namely for the CML-fit of the Poi-INAR(1) model at lag h=2, both for the refined asymptotics and the parametric bootstrap. The remaining model fits do not lead to a rejection, and one might ask, why? The reason seems to be the respective estimate of the AR(1)-parameter $\alpha_{1}=\rho \left(1\right)$, which equals only 0.396 for CML, but 0.452 for MM and 0.434 for semi-CML. So the CML-fit explains less of the dependency in the data. The deeper reason for this ambiguous outcome seems to be the low sample size n=96; according to Section 6, we can generally expect only mild power values. It is again interesting to compare the different critical values. For the Poi-INAR(1) CML-fit, bootstrap and refined asymptotics lead to rather similar critical values, in agreement with our simulation results in Section 6, where a similar performance of both methods was observed. For the remaining estimation approaches, the bootstrap critical values tend to be more narrow than the asymptotic ones, especially at lags 1 and 2. The strongest “shrinkage” of the critical values is observed for h=1, which goes along with our findings in Section 6, where the asymptotic implementation lead to severe undersizing at lag 1, whereas the bootstrap approaches held the nominal level quite well. Furthermore, due to the narrower critical values, the MM and semi-CML bootstraps are also more powerful at lags 1 and 2.

## 8. Conclusions

In this paper, we considered PACF model diagnostics for AR-type count processes based on raw data and on Pearson residuals, respectively. At first, we illustrated the limitations of the widely used and well-known asymptotic distribution result (as well as some refinements thereof) for the sample PACF values. Then, we introduced appropriate bootstrap schemes for the approximation of the correct sample PACF distribution. We considered a fully parametric bootstrap combined with MM and CML estimation, a semi-parametric bootstrap combined with CML estimation, and a fully non-parametric bootstrap scheme. We compared the performance of the different procedures for first- and second-order AR-type count processes. In the case where we apply the PACF test directly to the raw count data, the best performance was observed for the MM-based parametric bootstrap, CML-based semi-parametric bootstrap, and the refined asymptotic results, where the latter are preferable for computing time reasons. By contrast, when applying the PACF test to the Pearson residuals, we advise using the MM-based parametric bootstrap procedure which simultaneously provides good size properties and power performance. Finally, we applied our different PACF procedures to a well-known data set on claims counts and found some evidence for a higher-order model.

## Figures and Tables

**Table 1:** Rejection rates of PACF-tests applied to DGP with μ=5 and ρ(1)=0.5, where semi-parametric (parametric) bootstrap relies on null of (Poi-)INAR(1) process.

True DGP:		Poi-INAR(1)				NB-INAR(1),$\frac{\sigma^{2}}{\mu }=1.5$				Poi-INARCH(1)			
		PACF at Lag h=				PACF at Lag h=				PACF at Lag h=			
**Method**	n	**1**	**2**	**3**	**4**	**1**	**2**	**3**	**4**	**1**	**2**	**3**	**4**
asym.	100	0.998	0.053	0.040	0.044	0.998	0.054	0.043	0.046	0.995	0.054	0.046	0.044
(5)	1000	1.000	0.056	0.047	0.049	1.000	0.057	0.051	0.053	1.000	0.056	0.053	0.050
asym.	100	0.998	0.054	0.047	0.048	0.997	0.052	0.050	0.051	0.997	0.047	0.049	0.048
(11)	1000	1.000	0.050	0.049	0.051	1.000	0.061	0.053	0.049	1.000	0.060	0.051	0.048
param.	100	1.000	0.052	0.055	0.056	0.999	0.053	0.052	0.055	1.000	0.048	0.053	0.049
MM	1000	1.000	0.055	0.052	0.049	1.000	0.047	0.052	0.046	1.000	0.046	0.056	0.046
param.	100	1.000	0.054	0.051	0.054	0.999	0.049	0.053	0.050	0.999	0.059	0.046	0.049
CML	1000	1.000	0.048	0.049	0.057	1.000	0.050	0.055	0.051	1.000	0.052	0.052	0.047
semi-p.	100	1.000	0.053	0.053	0.051	1.000	0.054	0.051	0.049	0.999	0.044	0.048	0.054
CML	1000	1.000	0.047	0.054	0.054	1.000	0.051	0.049	0.057	1.000	0.052	0.054	0.052

**Table 2:** Like Table 1, but 2nd-order DGPs with $\alpha_{2}=0.2$.

True DGP:		Poi-INAR(2)				NB-INAR(2), $\frac{\sigma^{2}}{\mu }=1.5$				Poi-INARCH(2)			
		PACF at Lag h=				PACF at Lag h=				PACF at Lag h=			
**Method**	n	**1**	**2**	**3**	**4**	**1**	**2**	**3**	**4**	**1**	**2**	**3**	**4**
asym.	100	0.984	0.383	0.048	0.047	0.983	0.390	0.047	0.048	0.983	0.384	0.049	0.048
(5)	1000	1.000	1.000	0.053	0.053	1.000	1.000	0.056	0.052	1.000	1.000	0.055	0.053
asym.	100	0.990	0.478	0.048	0.049	0.987	0.480	0.053	0.047	0.986	0.480	0.053	0.054
(11)	1000	1.000	1.000	0.052	0.051	1.000	1.000	0.056	0.051	1.000	1.000	0.056	0.062
param.	100	0.996	0.470	0.055	0.058	0.995	0.465	0.053	0.054	0.995	0.482	0.048	0.054
MM	1000	1.000	1.000	0.056	0.058	1.000	1.000	0.053	0.051	1.000	1.000	0.055	0.054
param.	100	0.995	0.470	0.044	0.050	0.994	0.465	0.054	0.051	0.994	0.471	0.052	0.054
CML	1000	1.000	1.000	0.052	0.053	1.000	1.000	0.051	0.057	1.000	1.000	0.055	0.058
semi-p.	100	0.995	0.475	0.055	0.054	0.996	0.472	0.047	0.053	0.994	0.485	0.049	0.058
CML	1000	1.000	1.000	0.054	0.052	1.000	1.000	0.054	0.056	1.000	1.000	0.054	0.053

**Table 3:** Rejection rates of PACF-tests applied to DGP with μ=5, ρ(1)=0.5 and $\alpha_{2}=0.2$, where semi-parametric (parametric) bootstrap relies on null of (Poi-)INAR(2) process.

True DGP:		Poi-INAR(2)				NB-INAR(2), $\frac{\sigma^{2}}{\mu }=1.5$				Poi-INARCH(2)			
		PACF at Lag h=				PACF at Lag h=				PACF at Lag h=			
**Method**	n	**1**	**2**	**3**	**4**	**1**	**2**	**3**	**4**	**1**	**2**	**3**	**4**
asym.	100	0.984	0.383	0.048	0.047	0.983	0.390	0.047	0.048	0.983	0.384	0.049	0.048
(5)	1000	1.000	1.000	0.053	0.053	1.000	1.000	0.056	0.052	1.000	1.000	0.055	0.053
asym.	100	0.990	0.478	0.048	0.049	0.987	0.480	0.053	0.047	0.986	0.480	0.053	0.054
(11)	1000	1.000	1.000	0.052	0.051	1.000	1.000	0.056	0.051	1.000	1.000	0.056	0.062
param.	100	0.992	0.510	0.044	0.050	0.992	0.516	0.049	0.048	0.992	0.531	0.054	0.056
MM	1000	1.000	1.000	0.046	0.051	1.000	1.000	0.055	0.054	1.000	1.000	0.058	0.052
param.	100	0.977	0.447	0.057	0.048	0.994	0.478	0.052	0.050	0.991	0.446	0.055	0.056
CML	1000	1.000	1.000	0.050	0.053	1.000	1.000	0.053	0.051	1.000	1.000	0.054	0.051
semi-p.	100	0.993	0.548	0.053	0.047	0.990	0.521	0.053	0.054	0.992	0.498	0.051	0.049
CML	1000	1.000	1.000	0.055	0.047	1.000	1.000	0.049	0.047	1.000	1.000	0.051	0.051

**Table 10:** SPACF(h) of claims counts (bold font), lower and upper critical values (level 0.05) by different methods, where italic font indicates that critical value is violated.

	Lag *h*:	1	2	3	4	5
Upper	asym. (5)	*0.200*	0.200	0.200	0.200	0.200
critical value	asym. (11)	*0.186*	*0.175*	0.184	0.173	0.182
by method …	param., MM	*0.134*	*0.175*	0.184	0.181	0.186
	param., CML	*0.148*	*0.167*	0.179	0.182	0.185
	semi-p., CML	*0.138*	*0.171*	0.166	0.162	0.192
	SPACF(h)	**0.452**	**0.198**	**−0.010**	**−0.038**	**0.040**
Lower	asym. (5)	−0.200	−0.200	−0.200	−0.200	−0.200
critical value	asym. (11)	−0.207	−0.217	−0.205	−0.215	−0.203
by method …	param., MM	−0.211	−0.224	−0.206	−0.202	−0.199
	param., CML	−0.224	−0.223	−0.218	−0.204	−0.197
	semi-p., CML	−0.213	−0.213	−0.198	−0.220	−0.199

**Table 11:** SPACF(h) of Pearson residuals after fitting a (Poi-)INAR(1) model to the claims counts (bold font). Lower and upper critical values (level 0.05) by different methods, where italic font indicates that critical value is violated.

Poi-INAR(1), MM	Lag *h*:	1	2	3	4	5
Upper	asym. (5)	0.201	0.201	0.201	0.201	0.201
critical value	asym. (11)	0.187	0.176	0.185	0.174	0.183
by method …	param., MM	0.108	0.167	0.195	0.184	0.189
	SPACF(h)	**−0.060**	**0.156**	**0.061**	**−0.032**	**−0.007**
Lower	asym. (5)	−0.201	−0.201	−0.201	−0.201	−0.201
critical value	asym. (11)	−0.208	−0.218	−0.206	−0.216	−0.205
by method …	param., MM	−0.076	−0.190	−0.195	−0.195	−0.202
Poi-INAR(1), CML	Lag *h*:	1	2	3	4	5
Upper	asym. (5)	0.201	0.201	0.201	0.201	0.201
critical value	asym. (11)	0.187	*0.176*	0.185	0.174	0.183
by method …	param., CML	0.172	*0.166*	0.183	0.180	0.193
	SPACF(h)	**0.009**	**0.185**	**0.062**	**−0.031**	**−0.002**
Lower	asym. (5)	−0.201	−0.201	−0.201	−0.201	−0.201
critical value	asym. (11)	−0.208	−0.218	−0.206	−0.216	−0.205
by method …	param., CML	−0.208	−0.213	−0.219	−0.205	−0.201
INAR(1), semi-CML	Lag *h*:	1	2	3	4	5
Upper	asym. (5)	0.201	0.201	0.201	0.201	0.201
critical value	asym. (11)	0.187	0.176	0.185	0.174	0.183
by method …	semi-p., CML	0.158	0.171	0.178	0.162	0.203
	SPACF(h)	**−0.041**	**0.165**	**0.064**	**−0.029**	**−0.006**
Lower	asym. (5)	−0.201	−0.201	−0.201	−0.201	−0.201
critical value	asym. (11)	−0.208	−0.218	−0.206	−0.216	−0.205
by method …	semi-p., CML	−0.142	−0.204	−0.196	−0.215	−0.210

## Data Availability

The data presented in this study are available in the supplementary material.

## Supplementary Materials

The following supporting information can be downloaded at: https://www.mdpi.com/article/10.3390/e25010105/s1.

## Appendix A. On the Equivalence of ACF, PACF, and AR Coefficients

The first p Yule–Walker (YW) equations in

(A1) $$\rho \left(h\right)\;=\;\sum_{i=1}^{p}\;\alpha_{i}\;\rho \left(|h-i|\right)\;\mathrm{for}\;h=1,2,\ldots$$

can be rewritten in vector-matrix notation as follows: For k∈N, let $\alpha_{k}:={\left(\alpha_{1},\ldots ,\alpha_{k}\right)}^{⊤}\in R^{k}$ with $\alpha_{i}=0$ for i>p, let $r_{k}:={\left(\rho (1),\ldots ,\rho (k)\right)}^{⊤}\in R^{k}$, and

(A2) $$R_{k}\;:=\;{\left(\rho \left(|i-j|\right)\right)}_{i,j=1,\ldots ,k}\;=\;\left(\begin{array}{cccc} 1 & \rho (1) & \cdots & \rho (k-1)\\ \rho (1) & 1 & \ddots & \vdots \\ \vdots & \ddots & \ddots & \rho (1)\\ \rho (k-1) & \cdots & \rho (1) & 1 \end{array}\right)\;\in R^{k\times k}.$$

Then, (A1) implies that the AR(p) process satisfies the linear equation

(A3) $$R_{p}\;\alpha_{p}\;=\;r_{p}.$$

Note that $R_{k}$ constitutes a symmetric Toeplitz matrix, i.e., it is characterized by having constant diagonals. This type of matrix structure was first considered by Toeplitz [31,32], and it is crucial for efficiently solving (A3) in $\alpha_{p}$ (see the details below).

Assume that $R_{k}$ is invertible, and let $a_{k}\in R^{k}$ be the unique solution of the equation

(A4) $$R_{k}\;a_{k}\;=\;r_{k},\;i.e.,\;\;a_{k}\;=\;R_{k}^{-1}\;r_{k}.$$

Then, the PACF at lag *k* is defined by $\rho_{\mathrm{part}}\left(k\right):=a_{k,k}$ (last component of $a_{k}$); let us denote $\pi_{k}:={\left(\rho_{part}\left(1\right),\ldots ,\rho_{part}\left(k\right)\right)}^{⊤}\in R^{k}$.

If ${\left(X_{t}\right)}_{Z}$ follows an AR(p) model, then (A3) implies that

(A5) $$\rho_{\mathrm{part}}\left(p\right)\;=\;\alpha_{p},\;\rho_{\mathrm{part}}\left(h\right)\;=\;0\;\mathrm{for}\;\mathrm{all}\;h>p,$$

holds; in particular, we have $a_{p}=\alpha_{p}$. Because of the Toeplitz structure of $R_{k}$, the YW-equations (A3) can be solved recursively for k=1,2,…, which was first recognized by Durbin [33], Levinson [34]. The recursive scheme, which is commonly referred to as the *Durbin–Levinson (DL) algorithm*, can be expressed as

(A6) $$\begin{array}{cc} a_{k+1,k+1}\;= & \frac{\rho \left(k+1\right)\;-\;\sum_{i=1}^{k}a_{k,i}\;\rho \left(k+1-i\right)}{1\;-\;\sum_{i=1}^{k}a_{k,i}\;\rho \left(i\right)},\\ \left(\begin{array}{c} a_{k+1,1}\\ \vdots \\ a_{k+1,k} \end{array}\right)\;= & \left(\begin{array}{c} a_{k,1}\\ \vdots \\ a_{k,k} \end{array}\right)\;-\;a_{k+1,k+1}\left(\begin{array}{c} a_{k,k}\\ \vdots \\ a_{k,1} \end{array}\right). \end{array}$$

Given the (sample) ACF, the DL-algorithm (A6) is used to recursively compute the (sample) PACF for k=1,2,…, where $\rho_{\mathrm{part}}\left(1\right)=a_{1,1}=\rho \left(1\right)$.

Furthermore, applying the DL-algorithm to (A3), we can compute the AR parameters $\alpha_{1},\ldots ,\alpha_{p}$ corresponding to the ACF values ρ(1),…,ρ(p) (or if using the sample ACF, we end up with moment estimates for the AR parameters, referred to as *YW-estimates*). In R, this is readily implemented via acf2AR. Given the AR parameters, in turn, (A1) or (A3) can also be solved in the ACF, see Section 3.3 in Brockwell & Davis [11] as well as the R command ARMAacf.

The previous discussion shows that an AR(p) model can be characterized equivalently by either $\alpha_{p}$ or $r_{p}$. According to Barndorff-Nielsen & Schou [35], this type of “equivalent parametrization” can be further extended by the one-to-one relationship between $\alpha_{p}$ and $\pi_{p}$, i.e., we have one-to-one relations between $r_{p}↔\alpha_{p}↔\pi_{p}$. For computing $\alpha_{p}$ from $\pi_{p}$, Barndorff-Nielsen & Schou [35] suggest to use the DL-algorithm (A6) together with (A4) as follows:

(A7) $$\left(\begin{array}{c} a_{k+1,1}\\ \vdots \\ a_{k+1,k}\\ a_{k+1,k+1} \end{array}\right)\;=\;\left(\begin{array}{c} a_{k,1}\\ \vdots \\ a_{k,k}\\ 0 \end{array}\right)\;-\;\rho_{\mathrm{part}}\left(k+1\right)\left(\begin{array}{c} a_{k,k}\\ \vdots \\ a_{k,1}\\ -1 \end{array}\right)\;\mathrm{for}\;k=1,2,\ldots ,$$

which is initialized by setting $a_{1,1}=\rho_{\mathrm{part}}\left(1\right)$. Then, $\alpha_{p}=a_{p}$. Altogether, the application of the DL-algorithm allows the transformations

$$\begin{array}{ccc} r_{p}\; & \overset{\;(A3)\;}{\to } & \;\alpha_{p}\\ (A6)\;↘ & & ↗\;(A7)\\ & \pi_{p} & \end{array}$$

By contrast, recall that $\alpha_{p}\to r_{p}$ (and thus $\pi_{p}\to \alpha_{p}\to r_{p}$) is done by solving (A1) or (A3) in the ACF, e.g., by using the “third method” in Brockwell & Davis [11] (Section 3.3) or R’s ARMAacf.

## Appendix B. Further Simulation Results

**Table A1:** Rejection rates of PACF-test applied to DGP with μ=5, where semi-parametric (parametric) bootstrap relies on null of (Poi-)INAR(1) process.

True DGP:			Poi-INAR(1)				NB-INAR(1), $\frac{\sigma^{2}}{\mu }=1.5$				Poi-INARCH(1)			
			PACF at Lag h=				PACF at Lag h=				PACF at Lag h=			
**Method**	ρ(1)	n	**1**	**2**	**3**	**4**	**1**	**2**	**3**	**4**	**1**	**2**	**3**	**4**
asym.	0.25	100	0.629	0.050	0.047	0.043	0.646	0.050	0.042	0.044	0.639	0.049	0.046	0.045
(1.5)		1000	1.000	0.049	0.050	0.046	1.000	0.054	0.052	0.049	1.000	0.054	0.047	0.050
	0.5	100	0.998	0.053	0.040	0.044	0.998	0.054	0.043	0.046	0.995	0.054	0.046	0.044
		1000	1.000	0.056	0.047	0.049	1.000	0.057	0.051	0.053	1.000	0.056	0.053	0.050
	0.75	100	1.000	0.050	0.042	0.043	1.000	0.057	0.048	0.045	1.000	0.062	0.053	0.047
		1000	1.000	0.050	0.048	0.054	1.000	0.060	0.056	0.055	1.000	0.071	0.063	0.061
asym.	0.25	100	0.692	0.045	0.046	0.054	0.688	0.051	0.048	0.052	0.690	0.054	0.052	0.047
(3.2)		1000	1.000	0.053	0.050	0.052	1.000	0.053	0.051	0.047	1.000	0.051	0.050	0.049
	0.5	100	0.998	0.054	0.047	0.048	0.997	0.052	0.050	0.051	0.997	0.047	0.049	0.048
		1000	1.000	0.050	0.049	0.051	1.000	0.061	0.053	0.049	1.000	0.060	0.051	0.048
	0.75	100	1.000	0.047	0.051	0.046	1.000	0.054	0.054	0.050	1.000	0.060	0.061	0.053
		1000	1.000	0.055	0.054	0.054	1.000	0.062	0.058	0.056	1.000	0.073	0.066	0.060
param.	0.25	100	0.742	0.046	0.046	0.050	0.736	0.045	0.054	0.051	0.747	0.053	0.048	0.048
MM		1000	1.000	0.054	0.048	0.043	1.000	0.048	0.055	0.054	1.000	0.050	0.048	0.050
	0.5	100	1.000	0.052	0.055	0.056	0.999	0.053	0.052	0.055	1.000	0.048	0.053	0.049
		1000	1.000	0.055	0.052	0.049	1.000	0.047	0.052	0.046	1.000	0.046	0.056	0.046
	0.75	100	1.000	0.050	0.057	0.056	1.000	0.052	0.047	0.047	1.000	0.064	0.067	0.061
		1000	1.000	0.061	0.049	0.049	1.000	0.060	0.059	0.054	1.000	0.067	0.062	0.059
param.	0.25	100	0.747	0.049	0.049	0.054	0.735	0.046	0.049	0.051	0.726	0.053	0.052	0.055
CML		1000	1.000	0.051	0.049	0.047	1.000	0.049	0.058	0.049	1.000	0.044	0.051	0.048
	0.5	100	1.000	0.054	0.051	0.054	0.999	0.049	0.053	0.050	0.999	0.059	0.046	0.049
		1000	1.000	0.048	0.049	0.057	1.000	0.050	0.055	0.051	1.000	0.052	0.052	0.047
	0.75	100	1.000	0.052	0.050	0.051	1.000	0.051	0.051	0.050	1.000	0.061	0.055	0.053
		1000	1.000	0.050	0.050	0.052	1.000	0.052	0.058	0.053	1.000	0.069	0.066	0.066
semi-p.	0.25	100	0.736	0.043	0.046	0.048	0.723	0.049	0.052	0.051	0.733	0.053	0.053	0.056
CML		1000	1.000	0.048	0.052	0.047	1.000	0.046	0.053	0.057	1.000	0.054	0.059	0.046
	0.5	100	1.000	0.053	0.053	0.051	1.000	0.054	0.051	0.049	0.999	0.044	0.048	0.054
		1000	1.000	0.047	0.054	0.054	1.000	0.051	0.049	0.057	1.000	0.052	0.054	0.052
	0.75	100	1.000	0.052	0.054	0.054	1.000	0.051	0.051	0.041	1.000	0.064	0.063	0.057
		1000	1.000	0.048	0.046	0.051	1.000	0.051	0.048	0.048	1.000	0.060	0.057	0.060

**Table A2:** Rejection rates of PACF-test applied to Poi-INAR(1) DGP with μ=5, where circular block bootstrap with automatically selected (“b.star”) or fixed block length *b* is used.

			PACF at Lag h=					
Method	ρ(1)	*n*	1	2	3	4	5	6
b.star	0.25	100	0.922	0.010	0.006	0.007	0.007	0.007
		1000	1.000	0.037	0.025	0.016	0.011	0.006
	0.5	100	1.000	0.024	0.012	0.008	0.008	0.007
		1000	1.000	0.050	0.041	0.032	0.032	0.023
	0.75	100	1.000	0.041	0.027	0.019	0.015	0.010
		1000	1.000	0.045	0.050	0.042	0.037	0.035
b=5	0.25	100	0.857	0.031	0.024	0.011	0.005	0.005
		1000	1.000	0.038	0.023	0.011	0.003	0.004
	0.5	100	1.000	0.036	0.020	0.014	0.004	0.008
		1000	1.000	0.042	0.026	0.013	0.004	0.006
	0.75	100	1.000	0.028	0.021	0.008	0.002	0.008
		1000	1.000	0.125	0.074	0.038	0.016	0.029
b=10	0.25	100	0.833	0.052	0.047	0.036	0.026	0.022
		1000	1.000	0.045	0.041	0.036	0.031	0.025
	0.5	100	1.000	0.053	0.042	0.032	0.026	0.020
		1000	1.000	0.047	0.040	0.034	0.027	0.024
	0.75	100	1.000	0.045	0.041	0.035	0.023	0.020
		1000	1.000	0.054	0.040	0.034	0.029	0.019
b=15	0.25	100	0.831	0.058	0.050	0.051	0.037	0.031
		1000	1.000	0.043	0.048	0.045	0.037	0.030
	0.5	100	1.000	0.058	0.054	0.051	0.042	0.029
		1000	1.000	0.058	0.045	0.046	0.038	0.031
	0.75	100	1.000	0.054	0.042	0.035	0.036	0.034
		1000	1.000	0.053	0.044	0.039	0.031	0.036
b=20	0.25	100	0.813	0.064	0.060	0.055	0.051	0.047
		1000	1.000	0.053	0.046	0.045	0.046	0.041
	0.5	100	1.000	0.067	0.065	0.056	0.050	0.044
		1000	1.000	0.052	0.048	0.038	0.045	0.039
	0.75	100	1.000	0.059	0.064	0.051	0.043	0.041
		1000	1.000	0.049	0.048	0.047	0.034	0.038
b=25	0.25	100	0.819	0.080	0.072	0.058	0.058	0.056
		1000	1.000	0.054	0.058	0.051	0.047	0.044
	0.5	100	1.000	0.078	0.065	0.065	0.052	0.048
		1000	1.000	0.049	0.060	0.050	0.046	0.052
	0.75	100	1.000	0.070	0.064	0.058	0.048	0.051
		1000	1.000	0.054	0.050	0.050	0.048	0.040

**Table A3:** Rejection rates of PACF-test applied to Pearson residuals using MM estimates (DGPs with μ=5), where both residuals and parametric bootstrap rely on null of Poi-INAR(1) process.

True DGP:			Poi-INAR(1)				NB-INAR(1), $\frac{\sigma^{2}}{\mu }=1.5$				Poi-INARCH(1)			
			PACF at Lag h=				PACF at Lag h=				PACF at Lag h=			
**Method**	ρ(1)	n	**1**	**2**	**3**	**4**	**1**	**2**	**3**	**4**	**1**	**2**	**3**	**4**
asym.	0.25	100	0.000	0.038	0.044	0.047	0.000	0.041	0.040	0.046	0.000	0.042	0.046	0.046
(1.5)		1000	0.000	0.043	0.050	0.050	0.000	0.043	0.049	0.050	0.000	0.045	0.047	0.051
	0.5	100	0.000	0.026	0.040	0.043	0.001	0.026	0.038	0.044	0.000	0.031	0.041	0.044
		1000	0.000	0.029	0.044	0.053	0.000	0.030	0.043	0.052	0.001	0.033	0.044	0.050
	0.75	100	0.011	0.022	0.034	0.037	0.010	0.022	0.030	0.035	0.013	0.026	0.036	0.041
		1000	0.011	0.026	0.036	0.042	0.009	0.024	0.035	0.044	0.017	0.028	0.039	0.045
asym.	0.25	100	0.000	0.039	0.046	0.048	0.000	0.043	0.049	0.048	0.000	0.046	0.049	0.045
(3.2)		1000	0.000	0.046	0.052	0.055	0.000	0.044	0.048	0.048	0.000	0.047	0.047	0.053
	0.5	100	0.001	0.030	0.046	0.050	0.001	0.032	0.042	0.049	0.001	0.037	0.049	0.049
		1000	0.000	0.030	0.047	0.048	0.000	0.030	0.047	0.051	0.000	0.032	0.047	0.050
	0.75	100	0.014	0.031	0.038	0.047	0.015	0.030	0.037	0.045	0.019	0.036	0.045	0.053
		1000	0.011	0.026	0.036	0.042	0.010	0.024	0.034	0.043	0.018	0.029	0.040	0.045
param.	0.25	100	0.029	0.050	0.046	0.052	0.030	0.041	0.052	0.047	0.025	0.050	0.046	0.052
MM		1000	0.047	0.047	0.049	0.050	0.047	0.053	0.047	0.046	0.057	0.053	0.057	0.049
	0.5	100	0.056	0.051	0.052	0.048	0.050	0.049	0.049	0.045	0.066	0.050	0.047	0.051
		1000	0.051	0.053	0.049	0.045	0.050	0.051	0.053	0.051	0.060	0.046	0.050	0.044
	0.75	100	0.059	0.042	0.050	0.053	0.058	0.044	0.046	0.045	0.071	0.056	0.055	0.050
		1000	0.051	0.051	0.047	0.049	0.043	0.046	0.046	0.045	0.064	0.054	0.061	0.053

**Table A4:** Rejection rates of PACF-test applied to Pearson residuals using MM estimates (Poi-INAR(1) DGPs with μ=5), where circular block bootstrap with automatically selected (“b.star”) or fixed block length *b* is used.

			PACF at Lag h=					
Method	ρ(1)	*n*	1	2	3	4	5	6
Efron	0.25	100	0.000	0.047	0.046	0.047	0.051	0.049
		1000	0.000	0.040	0.047	0.051	0.048	0.046
	0.5	100	0.001	0.033	0.054	0.048	0.044	0.050
		1000	0.000	0.033	0.043	0.050	0.050	0.055
	0.75	100	0.014	0.030	0.041	0.044	0.047	0.053
		1000	0.011	0.028	0.032	0.044	0.043	0.046
b.star	0.25	100	0.000	0.041	0.041	0.048	0.045	0.054
		1000	0.000	0.049	0.052	0.047	0.049	0.053
	0.5	100	0.000	0.034	0.044	0.046	0.045	0.054
		1000	0.001	0.030	0.048	0.048	0.047	0.054
	0.75	100	0.008	0.037	0.040	0.044	0.044	0.049
		1000	0.007	0.027	0.033	0.041	0.044	0.049
b=5	0.25	100	0.000	0.024	0.035	0.048	0.044	0.047
		1000	0.000	0.023	0.035	0.044	0.055	0.053
	0.5	100	0.001	0.022	0.034	0.044	0.047	0.042
		1000	0.000	0.017	0.033	0.047	0.046	0.049
	0.75	100	0.003	0.017	0.035	0.042	0.050	0.047
		1000	0.003	0.013	0.028	0.039	0.044	0.051
b=10	0.25	100	0.000	0.015	0.019	0.027	0.032	0.035
		1000	0.000	0.010	0.017	0.025	0.028	0.033
	0.5	100	0.000	0.016	0.020	0.030	0.033	0.043
		1000	0.000	0.008	0.017	0.021	0.028	0.037
	0.75	100	0.003	0.012	0.018	0.023	0.030	0.034
		1000	0.002	0.008	0.013	0.020	0.025	0.035

**Table A5:** Rejection rates of PACF-test applied to Pearson residuals using CML estimates (DGPs with μ=5), where both residuals and bootstrap rely on null of Poi-INAR(1) process (parametric bootstrap) or unspecified INAR(1) process (semi-parametric bootstrap), respectively.

True DGP:			Poi-INAR(1)				NB-INAR(1), $\frac{\sigma^{2}}{\mu }=1.5$				Poi-INARCH(1)			
			PACF at Lag h=				PACF at Lag h=				PACF at Lag h=			
**Method**	ρ(1)	n	**1**	**2**	**3**	**4**	**1**	**2**	**3**	**4**	**1**	**2**	**3**	**4**
asym.	0.25	100	0.001	0.044	0.043	0.047	0.001	0.042	0.040	0.046	0.001	0.045	0.047	0.047
(1.5)		1000	0.000	0.045	0.051	0.052	0.236	0.057	0.049	0.050	0.000	0.048	0.048	0.052
	0.5	100	0.009	0.035	0.041	0.046	0.028	0.036	0.041	0.039	0.023	0.038	0.045	0.042
		1000	0.008	0.035	0.045	0.048	0.902	0.182	0.069	0.053	0.745	0.148	0.072	0.051
	0.75	100	0.032	0.042	0.043	0.042	0.057	0.041	0.044	0.040	0.364	0.113	0.070	0.047
		1000	0.033	0.040	0.048	0.050	0.581	0.288	0.162	0.094	1.000	0.913	0.488	0.204
asym.	0.25	100	0.001	0.043	0.047	0.049	0.002	0.049	0.050	0.048	0.000	0.046	0.049	0.049
(3.2)		1000	0.000	0.042	0.049	0.048	0.270	0.060	0.049	0.048	0.000	0.045	0.050	0.050
	0.5	100	0.009	0.041	0.046	0.048	0.043	0.055	0.047	0.050	0.034	0.053	0.048	0.051
		1000	0.009	0.039	0.046	0.053	0.909	0.198	0.077	0.058	0.753	0.167	0.072	0.055
	0.75	100	0.034	0.043	0.046	0.048	0.075	0.062	0.052	0.055	0.425	0.165	0.094	0.067
		1000	0.035	0.040	0.046	0.046	0.609	0.310	0.173	0.112	1.000	0.921	0.502	0.221
param.	0.25	100	0.040	0.052	0.049	0.048	0.262	0.050	0.051	0.056	0.046	0.051	0.051	0.056
CML		1000	0.049	0.046	0.053	0.049	1.000	0.065	0.051	0.054	0.194	0.047	0.050	0.047
	0.5	100	0.052	0.049	0.049	0.045	0.238	0.062	0.048	0.049	0.209	0.064	0.053	0.050
		1000	0.049	0.053	0.049	0.053	0.993	0.226	0.075	0.051	0.963	0.188	0.082	0.048
	0.75	100	0.046	0.046	0.050	0.051	0.123	0.079	0.054	0.052	0.606	0.190	0.097	0.068
		1000	0.048	0.053	0.048	0.047	0.709	0.338	0.169	0.121	1.000	0.936	0.522	0.221
semi-p.	0.25	100	0.037	0.045	0.047	0.050	0.049	0.044	0.050	0.054	0.045	0.051	0.052	0.051
CML		1000	0.037	0.054	0.050	0.047	0.037	0.047	0.052	0.052	0.031	0.051	0.054	0.054
	0.5	100	0.050	0.051	0.054	0.053	0.057	0.048	0.052	0.044	0.070	0.052	0.049	0.051
		1000	0.039	0.053	0.056	0.048	0.052	0.053	0.055	0.049	0.225	0.067	0.058	0.050
	0.75	100	0.051	0.044	0.049	0.050	0.049	0.049	0.046	0.047	0.223	0.104	0.072	0.061
		1000	0.046	0.049	0.049	0.051	0.055	0.053	0.050	0.050	0.377	0.217	0.129	0.080

**Table A6:** Rejection rates of PACF-test applied to Pearson residuals using CML estimates (Poi-INAR(1) DGPs with μ=5), where circular block bootstrap with automatically selected (“b.star”) or fixed block length *b* is used.

			PACF at Lag h=					
Method	ρ(1)	*n*	1	2	3	4	5	6
b.star	0.25	100	0.001	0.046	0.050	0.050	0.052	0.049
		1000	0.000	0.050	0.053	0.050	0.050	0.051
	0.5	100	0.007	0.036	0.046	0.054	0.045	0.052
		1000	0.005	0.034	0.046	0.047	0.050	0.050
	0.75	100	0.023	0.038	0.043	0.045	0.042	0.058
		1000	0.022	0.049	0.046	0.047	0.050	0.052
b=5	0.25	100	0.001	0.024	0.037	0.048	0.046	0.048
		1000	0.000	0.020	0.033	0.048	0.045	0.056
	0.5	100	0.003	0.020	0.038	0.039	0.050	0.050
		1000	0.002	0.018	0.035	0.045	0.051	0.049
	0.75	100	0.010	0.023	0.042	0.047	0.050	0.048
		1000	0.009	0.018	0.032	0.045	0.049	0.044
b=10	0.25	100	0.001	0.016	0.020	0.030	0.033	0.042
		1000	0.000	0.012	0.018	0.028	0.029	0.034
	0.5	100	0.003	0.014	0.019	0.030	0.031	0.036
		1000	0.001	0.010	0.015	0.020	0.027	0.031
	0.75	100	0.010	0.016	0.018	0.025	0.040	0.039
		1000	0.006	0.011	0.015	0.023	0.023	0.032
